# Supplementary material for: Diversity among Lasiodiplodia Species Causing Dieback, Root Rot and Leaf Spot on Fruit Trees in Egypt, and a Description of Lasiodiplodia newvalleyensis sp. nov
Source: J Fungi (Basel). 2022 Nov 15;8(11):1203. doi: 10.3390/jof8111203 (PMC9694705; doi:10.3390/jof8111203)
Supplement: Supplementary file 1 [file jof-08-01203-s001.zip › Table S1. Best fit model of evolution.pdf]

Table S1: Best-fit model of evolution according to BIC:
HKY+F+R2:part1+part3,HKY+F+I+I+R2:part2

ID	Model	LogL	AIC w-AIC	AICc w-AICc	BIC	w-BIC
**1**	HKY+F+R2	-2563.284	5140.568 + 1.05e-311	5140.703 - 6.95e-310	5173.702	4.43e-316
**2**	HKY+F+I+I+R2	-1607.577	3231.154 + 1.05e-311	3231.697 - 6.95e-310	3260.059	4.43e-316
