# Supplementary material for: Diversity among Lasiodiplodia Species Causing Dieback, Root Rot and Leaf Spot on Fruit Trees in Egypt, and a Description of Lasiodiplodia newvalleyensis sp. nov
Source: J Fungi (Basel). 2022 Nov 15;8(11):1203. doi: 10.3390/jof8111203 (PMC9694705; doi:10.3390/jof8111203)
Supplement: Supplementary file 1 [file jof-08-01203-s001.zip › Table S2. Isolates obtained in this study and their origins.pdf]

TableS2. Isolates obtained in this study and their origins

Isolate Number	Fungal identity	Host name	Plant organ	Origin
EGY2033	*L. laeliocattleyae*	*Citrus reticulata*	Twig	Beheira-Wady natron, Egypt
EGY2035	*L. theobromae*	*Citrus reticulata*	Leaf	Beheira-Wady natron, Egypt
EGY3036	*L. theobromae*	*Citrus reticulata*	Leaf	Giza-Abu Rawaash, Egypt
EGY2037	*L. theobromae*	*Citrus reticulata*	Leaf	Giza-Abu Rawaash, Egypt
EGY2038	*L. laeliocattleyae*	*Citrus reticulata*	Twig	Beheira-Wady natron, Egypt
EGY2041	*L. pseudotheobromae*	*Citrus sinensis*	Twig	Beheira-Nubaria, Egypt
EGY2042	*L. theobromae*	*Mangifera indica*	Stem cracking	Sharkia-ElMenayar, Egypt
EGY2043	*L. pseudotheobromae*	*Mangifera indica*	Branch	Beheira-Wady natron, Egypt
EGY2046	*L. theobromae*	*Pyrus communis*	Twig	Beheira-Alex desert road, Egypt
EGY2048	*L. pseudotheobromae*	*Prunus persica*	Root	Beheira-Wady natron, Egypt
EGY2049	*L. pseudotheobromae*	*Mangifera indica*	Branch	Beheira-Wady natron, Egypt
EGY2050	*L. theobromae*	*Pyrus communis*	Branch	Beheira-Alex desert road, Egypt
EGY2082	*L. theobromae*	*Mangifera indica*	Root	Kaliobyia, Egypt
EGY2083	*L. theobromae*	*Ficus carica*	Leaf	Beheira-Wady natron, Egypt
EGY20100	*L. theobromae*	*Prunus armeniaca*	Branch	Beheira-Wady natron, Egypt
EGY20101	*L. pseudotheobromae*	*Mangifera indica*	Leaf	Giza-Abu Rawaash, Egypt
EGY20113	*L. newvalleyensis*	*Phoenix dactylifera*	Leaf	New Valley-Southern Egypt, Egypt
EGY20114	*L. newvalleyensis*	*Phoenix dactylifera*	Leaf	New Valley-Southern Egypt, Egypt
