# Supplementary material for: Diversity among Lasiodiplodia Species Causing Dieback, Root Rot and Leaf Spot on Fruit Trees in Egypt, and a Description of Lasiodiplodia newvalleyensis sp. nov
Source: J Fungi (Basel). 2022 Nov 15;8(11):1203. doi: 10.3390/jof8111203 (PMC9694705; doi:10.3390/jof8111203)
Supplement: Supplementary file 1 [file jof-08-01203-s001.zip › Table S3. SNP positions of ITS, tef, and tub2 genes.pdf]

	ITS SNPs	
Consensus	GA-AAAGTTCAGAAGGTTCGTCCGGCGGGCGACGCCAACCGCTCCAAAGCGAGGTGTATT	59
Lasiodiplodia_newvalleyensis_EGY20114	..-................................-.......................	58
Lasiodiplodia_americana_CERC1961	..-..	59
Lasiodiplodia_exigua_BL104	..-..	59
Lasiodiplodia_caatinguensis_IBL366	..-..	59
Lasiodiplodia_macroconidia_GuoLD01752	..-....................................A...................	59
Lasiodiplodia_chonburiensis_MFLUCC160376	..-..	59
Lasiodiplodia_curvata_GuoLD01906	..-..	59
Lasiodiplodia_nanpingensis_CGMCC319596	..-..	59
Lasiodiplodia_irregularis_GuoLD01673	..-..	59
Lasiodiplodia_mahajangana_CMW27801	..-..	59
Lasiodiplodia_pandanicola_MFLUCC160265	-.G..	59
Lasiodiplodia_magnoliae_MFLUCC180948	..-..	59
Consensus	CTACTACGCTTGAGGGCTGAACAGCCACCGCCGAGGTCTTTGAGGCGCGTCCGCAGTGAG	119
Lasiodiplodia_newvalleyensis_EGY20114	....	118
Lasiodiplodia_americana_CERC1961	....	119
Lasiodiplodia_exigua_BL104	....	119
Lasiodiplodia_caatinguensis_IBL366	....	119
Lasiodiplodia_macroconidia_GuoLD01752	....	119
Lasiodiplodia_chonburiensis_MFLUCC160376	....	119
Lasiodiplodia_curvata_GuoLD01906	....	119
Lasiodiplodia_nanpingensis_CGMCC319596	....	119
Lasiodiplodia_irregularis_GuoLD01673	....	119
Lasiodiplodia_mahajangana_CMW27801	....	119
Lasiodiplodia_pandanicola_MFLUCC160265	....	119
Lasiodiplodia_magnoliae_MFLUCC180948	....	119
Consensus	GACGGTGCCCAATTCCAAGCAGAGCTTGAGGGTTGTAATGACGCTCGAACAGGCATGCCC	179
Lasiodiplodia_newvalleyensis_EGY20114	....	178
Lasiodiplodia_americana_CERC1961	....	179
Lasiodiplodia_exigua_BL104	....	179
Lasiodiplodia_caatinguensis_IBL366	....	179
Lasiodiplodia_macroconidia_GuoLD01752	....	179
Lasiodiplodia_chonburiensis_MFLUCC160376	....	179

Lasiodiplodia_curvata_GuoLD01906	....	179
Lasiodiplodia_nanpingensis_CGMCC319596	....	179
Lasiodiplodia_irregularis_GuoLD01673	....	179
Lasiodiplodia_mahajangana_CMW27801	....	179
Lasiodiplodia_pandanicola_MFLUCC160265	....	179
Lasiodiplodia_magnoliae_MFLUCC180948	....	179
Consensus	CCCGGAATACCAAGGGGCGCAATGTGCGTTCAAAGATTCGATGATTCACTGAATTCTGCA	239
Lasiodiplodia_newvalleyensis_EGY20114	....	238
Lasiodiplodia_americana_CERC1961	....	239
Lasiodiplodia_exigua_BL104	....	239
Lasiodiplodia_caatinguensis_IBL366	....	239
Lasiodiplodia_macroconidia_GuoLD01752	....	239
Lasiodiplodia_chonburiensis_MFLUCC160376	....	239
Lasiodiplodia_curvata_GuoLD01906	....	239
Lasiodiplodia_nanpingensis_CGMCC319596	....	239
Lasiodiplodia_irregularis_GuoLD01673	....	239
Lasiodiplodia_mahajangana_CMW27801	....	239
Lasiodiplodia_pandanicola_MFLUCC160265	....	239
Lasiodiplodia_magnoliae_MFLUCC180948	....	239
Consensus	ATTCACATTACTTATCGCATTTCGCTGCGTTCTTCATCGATGCCAGAACCAAGAGATCCG	299
Lasiodiplodia_newvalleyensis_EGY20114	....	298
Lasiodiplodia_americana_CERC1961	....	299
Lasiodiplodia_exigua_BL104	....	299
Lasiodiplodia_caatinguensis_IBL366	....	299
Lasiodiplodia_macroconidia_GuoLD01752	....	299
Lasiodiplodia_chonburiensis_MFLUCC160376	....	299
Lasiodiplodia_curvata_GuoLD01906	....	299
Lasiodiplodia_nanpingensis_CGMCC319596	....	299
Lasiodiplodia_irregularis_GuoLD01673	....	299
Lasiodiplodia_mahajangana_CMW27801	....	299
Lasiodiplodia_pandanicola_MFLUCC160265	....	299
Lasiodiplodia_magnoliae_MFLUCC180948	....	299
Consensus	TTGTTGAAAGTTTTAGTTTATTAACTTGTTTATCAGACGTCTGCGTTTACTGACTGGAGT	359
Lasiodiplodia_newvalleyensis_EGY20114	....	358

Lasiodiplodia_americana_CERC1961	....	359
Lasiodiplodia_exigua_BL104	....	359
Lasiodiplodia_caatinguensis_IBL366	....	359
Lasiodiplodia_macroconidia_GuoLD01752	....	359
Lasiodiplodia_chonburiensis_MFLUCC160376	....	359
Lasiodiplodia_curvata_GuoLD01906	....	359
Lasiodiplodia_nanpingensis_CGMCC319596	....	359
Lasiodiplodia_irregularis_GuoLD01673	....	359
Lasiodiplodia_mahajangana_CMW27801	....	359
Lasiodiplodia_pandanicola_MFLUCC160265	....	359
Lasiodiplodia_magnoliae_MFLUCC180948	....	359
Consensus	TTGAAGGTCCTTTGGCGGC--CGAAGCCGCCAAAGCAACAGAGGTACGTTCACAAAGGGT	417
Lasiodiplodia_newvalleyensis_EGY20114	..................--..G.....................................	416
Lasiodiplodia_americana_CERC1961	..................TT..--....................................	417
Lasiodiplodia_exigua_BL104	..................TT..--....................................	417
Lasiodiplodia_caatinguensis_IBL366	..................--..G.....................................	417
Lasiodiplodia_macroconidia_GuoLD01752	...G..............--..G.....................................	417
Lasiodiplodia_chonburiensis_MFLUCC160376	..................--..	417
Lasiodiplodia_curvata_GuoLD01906	..................--..	417
Lasiodiplodia_nanpingensis_CGMCC319596	..................--..	417
Lasiodiplodia_irregularis_GuoLD01673	..................--..	417
Lasiodiplodia_mahajangana_CMW27801	..................--..	417
Lasiodiplodia_pandanicola_MFLUCC160265	..................--..	417
Lasiodiplodia_magnoliae_MFLUCC180948	..................--..	417
Consensus	GGGAGAGTCGAGCCGAAGCT	437
Lasiodiplodia_newvalleyensis_EGY20114	...............G....	436
Lasiodiplodia_americana_CERC1961	....	437
Lasiodiplodia_exigua_BL104	....	437
Lasiodiplodia_caatinguensis_IBL366	....	437
Lasiodiplodia_macroconidia_GuoLD01752	C	437
Lasiodiplodia_chonburiensis_MFLUCC160376	....	437
Lasiodiplodia_curvata_GuoLD01906	....	437
Lasiodiplodia_nanpingensis_CGMCC319596	....	437
Lasiodiplodia_irregularis_GuoLD01673	....	437

Lasiodiplodia_mahajangana_CMW27801	....	437
Lasiodiplodia_pandanicola_MFLUCC160265	....	437
Lasiodiplodia_magnoliae_MFLUCC180948	C	437
	***tef1-a* SNPs**	
Consensus	CCCTTGCCGAGCTCAGCGGCTTCCTGTAGTGGGGCGCGTTAGCCTTTGCTCGTACGACGG	60
Lasiodiplodia_newvalleyensis_EGY20114	....	60
Lasiodiplodia_americana_CERC1961	.....A..	60
Lasiodiplodia_exigua_BL104	....	60
Lasiodiplodia_caatinguensis_IBL366	--------------------.....................T..................	40
Lasiodiplodia_macroconidia_GuoLD01752	.....A...................................T..................	60
Lasiodiplodia_chonburiensis_MFLUCC160376	.....A...................................T..................	60
Lasiodiplodia_curvata_GuoLD01906	....	60
Lasiodiplodia_nanpingensis_CGMCC319596	....	60
Lasiodiplodia_irregularis_GuoLD01673	....	60
Lasiodiplodia_mahajangana_CMW27801	....	60
Lasiodiplodia_pandanicola_MFLUCC160265	-----------.AA.TAA....T....TC..T............................	49
Lasiodiplodia_magnoliae_MFLUCC180948	----------------------------................................	32
Consensus	TACATGAGTGGTCAGAGCATTTTTCGCTAGTGGGGAGGCGCGTCGGCCAGACGCCGGTCG	120
Lasiodiplodia_newvalleyensis_EGY20114	....	120
Lasiodiplodia_americana_CERC1961	....	120
Lasiodiplodia_exigua_BL104	....	120
Lasiodiplodia_caatinguensis_IBL366	....	100
Lasiodiplodia_macroconidia_GuoLD01752	....	120
Lasiodiplodia_chonburiensis_MFLUCC160376	....	120
Lasiodiplodia_curvata_GuoLD01906	....	120
Lasiodiplodia_nanpingensis_CGMCC319596	....	120
Lasiodiplodia_irregularis_GuoLD01673	....	120
Lasiodiplodia_mahajangana_CMW27801	....	120
Lasiodiplodia_pandanicola_MFLUCC160265	....	109
Lasiodiplodia_magnoliae_MFLUCC180948	....	92
Consensus	CAAAAAAACCAAGTGCGGTCATTTTGCCGAACCCAGACGAGGCTAGCGCGGGCCAAACCC	180
Lasiodiplodia_newvalleyensis_EGY20114	....	180
Lasiodiplodia_americana_CERC1961	....	180
Lasiodiplodia_exigua_BL104	....	180

Lasiodiplodia_caatinguensis_IBL366	....	160
Lasiodiplodia_macroconidia_GuoLD01752	....	180
Lasiodiplodia_chonburiensis_MFLUCC160376	....	180
Lasiodiplodia_curvata_GuoLD01906	....	180
Lasiodiplodia_nanpingensis_CGMCC319596	....	180
Lasiodiplodia_irregularis_GuoLD01673	....	180
Lasiodiplodia_mahajangana_CMW27801	....	180
Lasiodiplodia_pandanicola_MFLUCC160265	....	169
Lasiodiplodia_magnoliae_MFLUCC180948	....	152
Consensus	CACCACGAAAAATGCCCCTCACCAAAGCGATAAGGCGGACTATGCGCC--------GCAG	232
Lasiodiplodia_newvalleyensis_EGY20114	....--------....	232
Lasiodiplodia_americana_CERC1961	....--------....	232
Lasiodiplodia_exigua_BL104	....--------....	232
Lasiodiplodia_caatinguensis_IBL366	....GCAGCGCC....	220
Lasiodiplodia_macroconidia_GuoLD01752	....GCAGCGCC....	240
Lasiodiplodia_chonburiensis_MFLUCC160376	....GCAGCGCC....	240
Lasiodiplodia_curvata_GuoLD01906	....GCAGCGCT....	240
Lasiodiplodia_nanpingensis_CGMCC319596	....--------....	232
Lasiodiplodia_irregularis_GuoLD01673	....--------....	232
Lasiodiplodia_mahajangana_CMW27801	....--------....	232
Lasiodiplodia_pandanicola_MFLUCC160265	....--------....	221
Lasiodiplodia_magnoliae_MFLUCC180948	....GCAGCGCC....	212
Consensus	CGCCGAAGTCGAGAGGGG	250
Lasiodiplodia_newvalleyensis_EGY20114	....	250
Lasiodiplodia_americana_CERC1961	....	250
Lasiodiplodia_exigua_BL104	....	250
Lasiodiplodia_caatinguensis_IBL366	....A.............	238
Lasiodiplodia_macroconidia_GuoLD01752	....A.............	258
Lasiodiplodia_chonburiensis_MFLUCC160376	....A............-	257
Lasiodiplodia_curvata_GuoLD01906	....A.............	258
Lasiodiplodia_nanpingensis_CGMCC319596	....A.............	250
Lasiodiplodia_irregularis_GuoLD01673	....	250
Lasiodiplodia_mahajangana_CMW27801	....	250
Lasiodiplodia_pandanicola_MFLUCC160265	....	239

Lasiodiplodia_magnoliae_MFLUCC180948	....	230
	***tub2* SNPs**	
Consensus	CTCCTGCTCCTGCGC-CCCCCGCTGACGGAAGCGACACCATAGGCAGACCATCTCTGGCG	59
Lasiodiplodia_newvalleyensis_EGY20114	............A.-...	59
Lasiodiplodia_americana_CERC1961	...............C..	60
Lasiodiplodia_exigua_BL104	...............-..	59
Lasiodiplodia_caatinguensis_IBL366	...............-..	59
Lasiodiplodia_macroconidia_GuoLD01752	...............-..	59
Lasiodiplodia_chonburiensis_MFLUCC160376	----------------------......................................	38
Lasiodiplodia_curvata_GuoLD01906	.........-.....-..	58
Lasiodiplodia_nanpingensis_CGMCC319596	...............-..	59
Lasiodiplodia_irregularis_GuoLD01673	...............-..	59
Lasiodiplodia_mahajangana_CMW27801	...............-..	59
Lasiodiplodia_magnoliae_MFLUCC180948	...............-...........-................................	58
Consensus	AGCACGGCCTGGATGGCTCCGGTGTGTAAGTGTGCGCCTTCTCCGCCGCGCATCGCAATC	119
Lasiodiplodia_newvalleyensis_EGY20114	....	119
Lasiodiplodia_americana_CERC1961	....	120
Lasiodiplodia_exigua_BL104	....	119
Lasiodiplodia_caatinguensis_IBL366	....	119
Lasiodiplodia_macroconidia_GuoLD01752	....G......	119
Lasiodiplodia_chonburiensis_MFLUCC160376	....	98
Lasiodiplodia_curvata_GuoLD01906	....	118
Lasiodiplodia_nanpingensis_CGMCC319596	....	119
Lasiodiplodia_irregularis_GuoLD01673	....	119
Lasiodiplodia_mahajangana_CMW27801	..................................T.........................	119
Lasiodiplodia_magnoliae_MFLUCC180948	....	118
Consensus	GCTGACCCGTAGCAGCTACAATGGCACCTCGGACCTCCAGCTGGAGCGCATGAACGTCTA	179
Lasiodiplodia_newvalleyensis_EGY20114	....	179
Lasiodiplodia_americana_CERC1961	....	180
Lasiodiplodia_exigua_BL104	....	179
Lasiodiplodia_caatinguensis_IBL366	....	179
Lasiodiplodia_macroconidia_GuoLD01752	....A....................	179
Lasiodiplodia_chonburiensis_MFLUCC160376	....	158
Lasiodiplodia_curvata_GuoLD01906	....	178

Lasiodiplodia_nanpingensis_CGMCC319596	....	179
Lasiodiplodia_irregularis_GuoLD01673	....	179
Lasiodiplodia_mahajangana_CMW27801	....	179
Lasiodiplodia_magnoliae_MFLUCC180948	....	178
Consensus	CTTCAACGAGGTACTCTCTCCATAATTAGACAAACACGTAAAGTATGGCAATCTTCTGAA	239
Lasiodiplodia_newvalleyensis_EGY20114	...................A..	239
Lasiodiplodia_americana_CERC1961	....	240
Lasiodiplodia_exigua_BL104	....	239
Lasiodiplodia_caatinguensis_IBL366	...................A..	239
Lasiodiplodia_macroconidia_GuoLD01752	....	239
Lasiodiplodia_chonburiensis_MFLUCC160376	....	218
Lasiodiplodia_curvata_GuoLD01906	....	238
Lasiodiplodia_nanpingensis_CGMCC319596	....	239
Lasiodiplodia_irregularis_GuoLD01673	....	239
Lasiodiplodia_mahajangana_CMW27801	..................................A.........................	239
Lasiodiplodia_magnoliae_MFLUCC180948	....	238
Consensus	CGCGCAGCAGGCGTCCAACAACAAGTACGTTCCTCGTGCTGTCCTCGTCGACCTCGAGCC	299
Lasiodiplodia_newvalleyensis_EGY20114	....	299
Lasiodiplodia_americana_CERC1961	....	300
Lasiodiplodia_exigua_BL104	....	299
Lasiodiplodia_caatinguensis_IBL366	....	299
Lasiodiplodia_macroconidia_GuoLD01752	....	299
Lasiodiplodia_chonburiensis_MFLUCC160376	....	278
Lasiodiplodia_curvata_GuoLD01906	....	298
Lasiodiplodia_nanpingensis_CGMCC319596	....	299
Lasiodiplodia_irregularis_GuoLD01673	....	299
Lasiodiplodia_mahajangana_CMW27801	....	299
Lasiodiplodia_magnoliae_MFLUCC180948	....	298
Consensus	CGGCACCATGGATGCCGTCCGCGCCGGCCCCTTCGGCCAGCTCTTCCGCCCCGACAA	356
Lasiodiplodia_newvalleyensis_EGY20114	....	356
Lasiodiplodia_americana_CERC1961	....	357
Lasiodiplodia_exigua_BL104	....	356
Lasiodiplodia_caatinguensis_IBL366	....	356
Lasiodiplodia_macroconidia_GuoLD01752	....	356

Lasiodiplodia_chonburiensis_MFLUCC160376	....	335
Lasiodiplodia_curvata_GuoLD01906	....	355
Lasiodiplodia_nanpingensis_CGMCC319596	....	356
Lasiodiplodia_irregularis_GuoLD01673	....	356
Lasiodiplodia_mahajangana_CMW27801	....	356
Lasiodiplodia_magnoliae_MFLUCC180948	...........................-....-------------------------	329
